# Variability in Paralytic Shellfish Toxin Profiles and Dinoflagellate Diversity in Mussels and Seawater Collected during Spring in Korean Coastal Seawater

**DOI:** 10.3390/toxins16080338

**Published:** 2024-07-31

**Authors:** Dong Han Choi, Wonseok Yang, Young-Eun Kim, Bum Soo Park, Jiyeon Sung, Jaeho Choi, Jung-Rae Rho, Young Seok Han, Yeonjung Lee

**Affiliations:** 1 Ocean Climate Response and Ecosystem Research Department, Korea Institute of Ocean Science and Technology, Busan 49111, Republic of Korea; dhchoi@kiost.ac.kr (D.H.C.); ywseok@kiost.ac.kr (W.Y.); 2 Department of Convergence Study on the Ocean Science and Technology, Ocean Science and Technology School, Korea Maritime and Ocean University, Busan 49112, Republic of Korea; 3 Environmental Measurement & Analysis Center, National Institute of Environmental Research, Incheon 22689, Republic of Korea; biot7212@gmail.com; 4 Department of Life Science, College of Natural Sciences, Hanyang University, Seoul 04763, Republic of Korea; parkbs@hanyang.ac.kr (B.S.P.); giyun.sung@stonybrook.edu (J.S.); 5 Hanyang Institute of Bioscience and Biotechnology, Hanyang University, Seoul 04763, Republic of Korea; 6 Research Institute for Natural Sciences, Hanyang University, Seoul 04763, Republic of Korea; 7 School of Earth and Environmental Sciences, Seoul National University, Seoul 08826, Republic of Korea; jhc92617@snu.ac.kr; 8 Research Institute of Oceanography, Seoul National University, Seoul 08826, Republic of Korea; 9 Department of Oceanography, Kunsan National University, Kunsan 54150, Republic of Korea; jrrho@kunsan.ac.kr; 10 NEB Company, Bucheon 14523, Republic of Korea; hanulva@neoenbiz.com

**Keywords:** paralytic shellfish poisoning (PSP), paralytic shellfish toxins (PST), dinoflagellate diversity, microalgae, *Alexandrium* species, mussel

## Abstract

Paralytic shellfish toxins (PSTs) are potent neurotoxins produced by certain microalgae, particularly dinoflagellates, and they can accumulate in shellfish in coastal seawater and thus pose significant health risks to humans. To explore the relationship between toxicity and PST profiles in seawater and mussels, the spatiotemporal variations in PST concentrations and profiles were investigated along the southern coast of Korea under peak PST levels during spring. Seawater and mussel samples were collected biweekly from multiple stations, and the toxin concentrations in the samples were measured. Moreover, the dinoflagellate community composition was analyzed using next-generation sequencing to identify potential PST-producing species. The PST concentrations and toxin profiles showed substantial spatiotemporal variability, with GTX1 and GTX4 representing the dominant toxins in both samples, and C1/2 tending to be higher in seawater. *Alexandrium* species were identified as the primary sources of PSTs. Environmental factors such as water temperature and salinity influenced PST production. This study demonstrates that variability in the amount and composition of PSTs is due to intricate ecological interactions. To mitigate shellfish poisoning, continuous monitoring must be conducted to gain a deeper understanding of these interactions.

## 1. Introduction

Paralytic shellfish toxins (PST) are a group of potent neurotoxins that can accumulate in the tissues of certain shellfish, such as mussels, clams, and oysters [1,2]. When consumed by humans, PSTs can cause a range of symptoms, including tingling, numbness, dizziness, and respiratory paralysis. In severe cases, PST poisoning can be fatal [3,4]. PSTs are produced by certain types of microalgae that bloom in coastal waters and are ingested by filter-feeding shellfish [4]. Shellfish can accumulate these toxins in their tissues without being affected, making them potentially dangerous for human consumption. The severity of PST contamination in shellfish is dependent upon toxic microalgae concentrations in the water as well as the duration that the shellfish have been feeding on them.

PSTs are produced by diverse marine microalgae, particularly dinoflagellates, which are capable of producing these toxins as a defense mechanism [5,6]. Several species of dinoflagellates are known to produce PSTs in seawater, such as *Alexandrium* spp., *Pyrodinium bahamense*, *Pyrodinium* spp., and *Gymnodinium catenatum* [4]. The *Alexandrium* spp. are among the most well-known and widely distributed genera of dinoflagellates that produce PSTs in temperate coastal waters. In East Asia, *Alexandrium* spp. blooms have been reported in several regions, including Japan, Korea, and China [7,8,9]. In addition to these regions, *Alexandrium* blooms have been reported in other temperate coastal waters worldwide, including those in Europe and North America [10,11]. Furthermore, there have been several reports of paralytic shellfish poisoning (PSP) outbreaks caused by microalgae other than *Alexandrium* in different regions of the world. In Mexico and El Salvador, *Pyrodinium bahamense* blooms have been linked to PSP in humans and marine wildlife, including dolphins and sea turtles [12,13]. *Gymnodinium catenatum* blooms have been identified as the main cause of PSP in humans, with several outbreaks reported in recent years [14,15]. PSTs can be produced by a variety of freshwater or brackish cyanobacteria, *Anabaena circinalis*, *A. lemmermannii*, *Aphanizomenon gracile*, *A. issatschenkoi*, *Cylindrospermopsis raciborskii*, *Lyngbya wollei*, *Planktothrix* spp., and *Rivularia* spp. [16]. Numerous microalgal species have been known to produce PSTs.

The frequency and severity of these blooms can vary depending on various environmental factors such as water temperature, nutrient availability, and ocean currents [13,17,18]. Thus, environmental factors play crucial roles in shaping the frequency and severity of harmful algal blooms, including those caused by *Alexandrium* and other PST-producing microalgae, resulting in temporal and spatial variations in PST concentrations.

Temporal variations in PST concentrations and profiles have been investigated in seawater and shellfish from temperate waters [19,20,21,22]. In Korean coastal waters, PSTs in mussels have been known to occur beginning in March, peaking in concentration at water temperatures around 15 °C and slowly disappearing from mid-June when sea temperatures reach 18 °C or higher [22,23]. This temporal pattern was generally consistent with the growth and decline of the *Alexandrium catenella* bloom. However, other *Alexandrium* species, namely, *A. tamarense* and *A. pacificum*, are known to produce PSTs [24,25]. Thus, *Alexandrium* has also been regarded as the main cause of PSP outbreaks in Korea. Furthermore, during the *Alexandrium* bloom, various PSTs with particle sizes smaller than 20 μm were also detected, which excludes *Alexandrium*. This raises the possibility that the toxins were produced by microalgae or cyanobacteria other than *Alexandrium* [21,26]. Significant discrepancies have frequently been observed between simultaneous measurements of *Alexandrium* abundance in the water column and the toxin content in shellfish [19,21]. Thus, it seems likely that PSTs are produced by other unknown microalgae species.

In this study, we investigated the toxin concentrations in microalgae in seawater and mussel samples at approximately 2-week intervals from winter to spring, which is the period when PST levels typically peak. In addition, the dinoflagellate community composition in seawater and mussel guts was analyzed using next-generation sequencing. This analysis aimed to reveal the temporal and spatial variability of PST profiles of microalgae and mussels, determine the interrelationship between toxins in microalgae and mussels, and identify the microalgae responsible for mussel poisoning.

## 2. Results

### 2.1. Seawater Temperature and Salinity Variations

**Figure 1:**
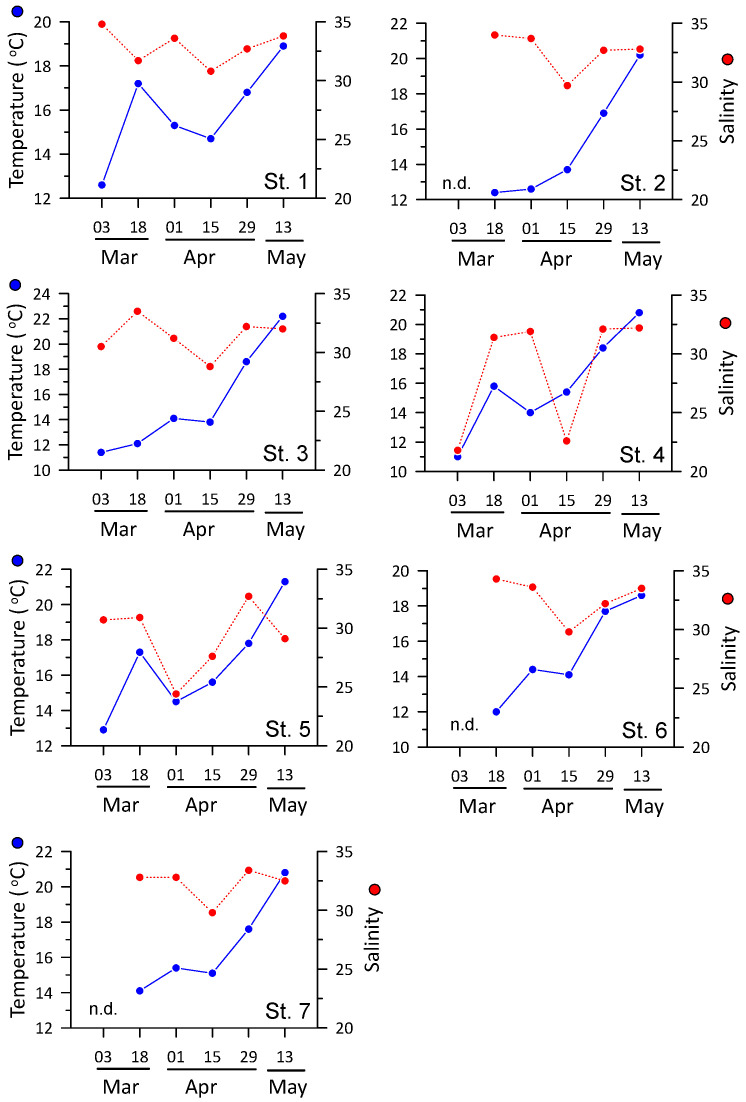
Temperature and salinity variations measured in the surface seawater of each station during the study period. “n.d.” means “no data”.

At the beginning of the survey in early March, water temperatures were low, at an average of 12.0 °C, although they increased to approximately 15 °C from mid-March and rose significantly to averages of 17.6 °C and 20.4 °C in late April and May, respectively (Figure 1). During each survey, the difference in water temperature between stations ranged from 2 to 5 °C, showing a relatively large spatial difference in water temperature (Figure 1). Mean salinity ranged from 28.4 to 32.8 in each survey period, while differences in salinity among stations varied from 1.3 to 13.0 in each survey, showing greater inter-station variability than temporal variability (Figure 1). In particular, low salinities of approximately 22 were observed at station (Stn) 4 in early March and mid-April, suggesting an intermittent influx of large volumes of freshwater from the surrounding land.

### 2.2. Concentration of PSTs in Seawater

**Figure 2:**
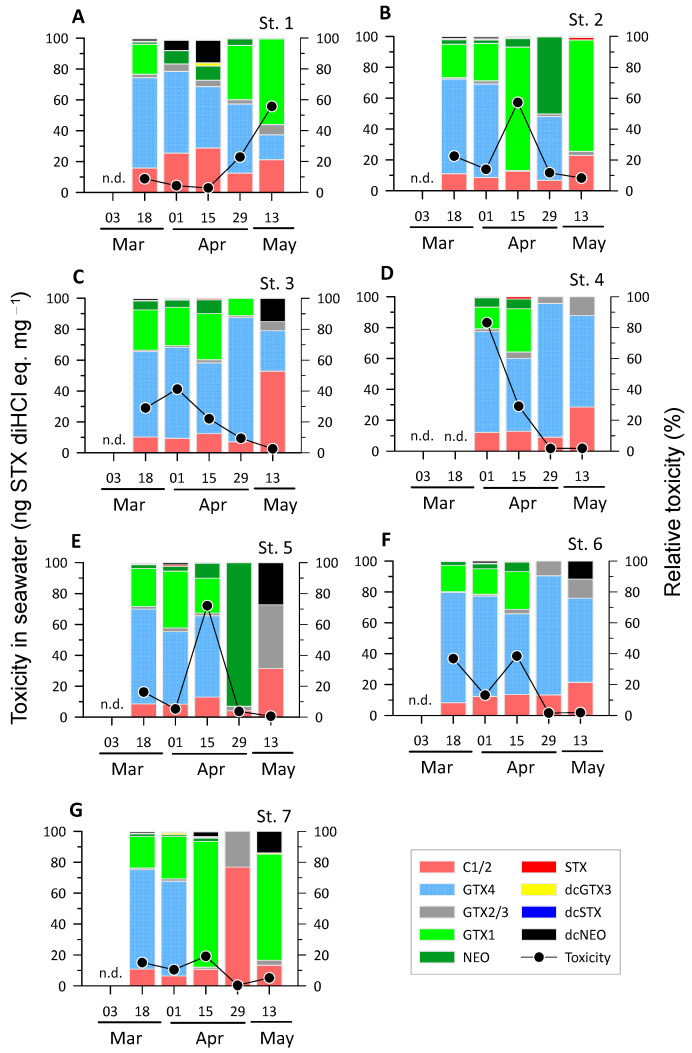
Toxicity and relative toxicity of paralytic shellfish toxins (PSTs) measured in seawater samples at seven different stations (**A**–**G**) for Stns 1–7 from March to May. The closed circles and colored bars show the total toxicity (left *Y*-axis) and the relative toxicity of each toxin (right *Y*-axis), respectively. “n.d.” indicates that the toxin content was not analyzed.

For microalgae collected using a 20 μm net, the PST toxicity showed a large variation ranging from 0.3 to 83.2 ng STX diHCl eq. mg^−1^ (eq. means toxicity equivalents) (Figure 2). Peaks in the range of 38.5–83.2 ng STX diHCl eq. mg^−1^ were observed at each station, with the exception of Stn 7, which was located outside the bay. This site exhibits PST toxicity as low as 19.1 ng STX diHCl eq. mg^−1^ or less. The maximum toxicities occurred in early and mid-April at most stations, but at Stn 1, the maximum value of 55.7 ng STX diHCl eq. mg^−1^ occurred in mid-May. Thus, the toxicity of PSTs in seawater showed large temporal and spatial variations in the study area.

The concentrations of the nine PSP toxins in each seawater sample were measured to determine the spatiotemporal variations in the toxin profile (Figure 2). In general, the highest toxicities were observed for C1/2, gonyautoxin (GTX)1, and GTX4, although neosaxitoxin (NEO), GTX2/3, and decabamoylneosaxitoxin (dcNEO) also showed high toxicity in several samples. Among the toxins, GTX4 was dominant in most of the samples (23 out of 34 samples), especially at the stations around the mussel farms of the inner bay (Stns 4–6). However, after mid-April, GTX4 was absent or showed decreased relative toxicity in some samples. Conversely, although GTX1 showed a relatively low proportion of less than 5% in mid-March, the proportion tended to increase significantly up to 82% in several samples after mid-April. Toxin C1/2 generally tended to increase over time, with a high occurrence of 77% at Stn 7 at the end of April. Toxin NEO was mostly distributed in a low proportion (less than 10%), but it occupied a high percentage of 50–93% in some samples with low total toxicity. Toxins dcNEO and GTX2/3 also presented relatively high proportions of 10–41% and 15–27%, respectively, in samples with a total toxicity of less than 2.5 ng STX diHCl eq. mg^−1^. STX (saxitoxin) toxicity occurred at a maximum of 1.5% or less and was a very minor toxin in the study area.

### 2.3. Concentration of PSTs in Mussels

**Figure 3:**
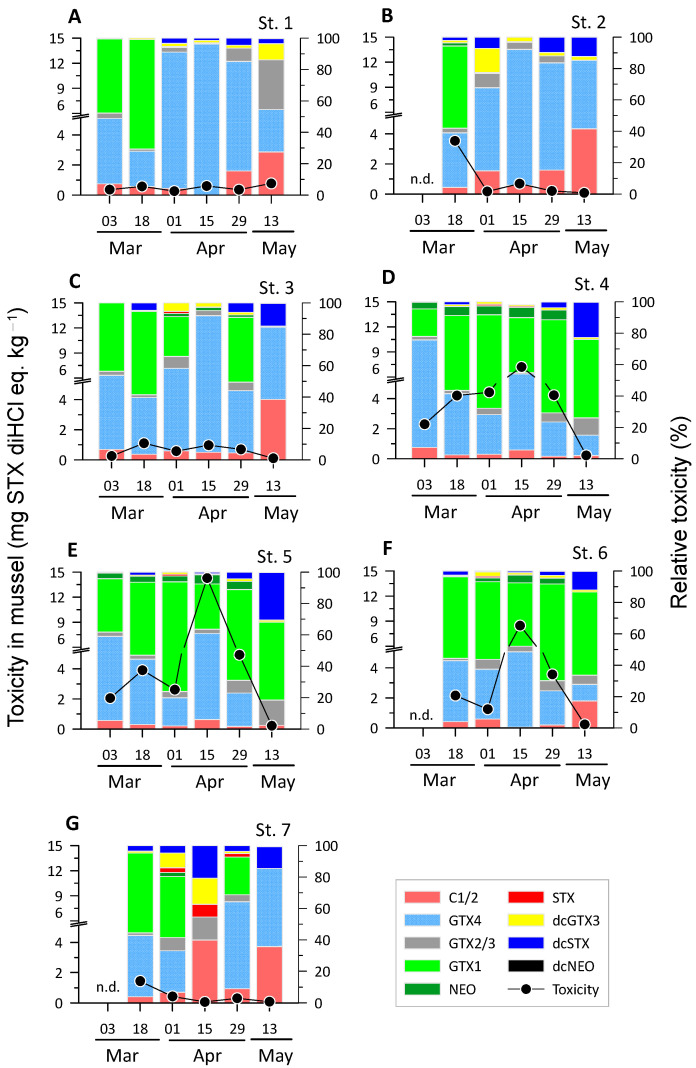
Toxicity and relative toxicity of paralytic shellfish toxins (PSTs) measured in mussels from seven different stations (**A**–**G**) for Stns 1–7 from March to May. The closed circles and colored bars show the total toxicity (left *Y*-axis) and the relative toxicity of each toxin (right *Y*-axis), respectively. “n.d.” indicates that the toxin content was not analyzed.

During the study period, the total toxicity of PSTs found in mussel tissues varied greatly among the samples, ranging from 0.1 to 14.3 mg STX diHCl eq. kg^−1^ (Figure 3). In particular, Stns 4, 5, and 6, situated in the inner bay where mussel farms are located, consistently showed high total PST toxicities above 1.3 mg STX diHCl eq. kg^−1^, except in mid-May. At the other stations, PST toxicities varied widely in the tissues of the mussel samples, with most being below 1.0 mg STX diHCl eq. kg^−1^, except for a few samples collected in mid-March. At the inner bay stations, most of the surveys showed toxicities above the Korean safety standards for PSP toxins (0.8 mg STX diHCl eq. kg^−1^), and the PST toxicity increased gradually from early March, peaked in mid-April, and then decreased to approximately 0.2 mg STX diHCl eq. kg^−1^ in mid-May.

Similar to the seawater samples, GTX1 and GTX4 were the dominant toxins in the mussel samples (Figure 3). In early spring, when toxin concentrations were relatively low at the inner bay stations, the proportion of GTX4 was generally high and then gradually decreased, while the proportion of GTX1 increased towards the end of the survey. However, in mid-April, when toxin concentrations were highest at each station, the proportions of GTX4 were somewhat higher or similar to the proportions of GTX1. In the inner bay stations (Stns 4–6), the dominant toxins in mussels and seawater were GTX1 and GTX4, respectively. Meanwhile, decabamoylsaxitoxin (dcSTX), which was not significant in the seawater samples, was present in several samples at a maximum of 30%. However, the toxicity of the PSTs in these samples was low at less than 0.2 mg STX diHCl eq. kg^−1^. Decabamoylgonyautoxin 3 (dcGTX3) did not exhibit a significant presence in seawater but was found at a high proportion of approximately 15% in the mussel samples. However, similar to dcSTX, dcGTX3 was relatively high in samples with low total PST toxicity. STX, which was not found in most of the seawater samples, was only present in a few samples (percentage of 8% or less) from Stn 7, where the total toxin concentrations were low, suggesting that STX was not a dominant toxin in this study area.

### 2.4. Dinoflagellate Composition in Seawater and Mussel Samples

**Figure 4:**
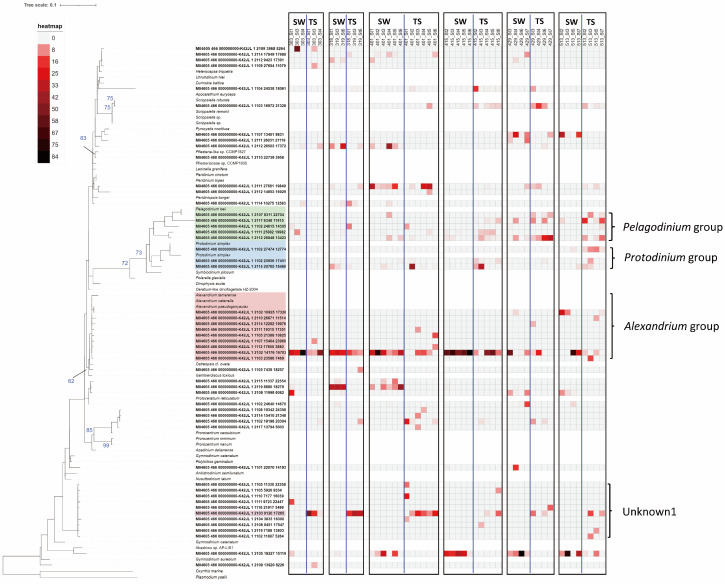
Phylogenetic tree and heatmap of *cob* gene sequences of dinoflagellates derived from seawater (SW) and tissue (TS) samples. Only the operational taxonomic units (OTUs) that accounted for more than 5% in at least one sample were included in the analysis. The colors in the heatmap represent the relative dominance of operational taxonomic units in each sample. The sample name (mdd_Stx) indicates “month–day_station”. The numbers in the phylogenetic tree represent the bootstrap values (100 resampling), and only values above 60% are shown in the tree.

In total, 153 operational taxonomic units (OTUs) accounted for more than 1% of the number of reads in at least one of the samples, and 54 OTUs accounted for more than 5% of the reads in at least one sample (Figure 4).

In most investigations, the *Alexandrium* group was dominant in both the seawater and mussel guts (Figure 4). However, the proportion of the *Alexandrium* group in seawater declined from late April, when the OTUs closely related to *Akashiwo* sp. were dominant in seawater, while those related to *Pelagodinium* and *Protodinium* were dominant in the mussel guts. Several other OTUs with relatively high percentages appeared in various spatiotemporal samples, indicating the presence of a large variety of dinoflagellates during the study period.

**Figure 5:**
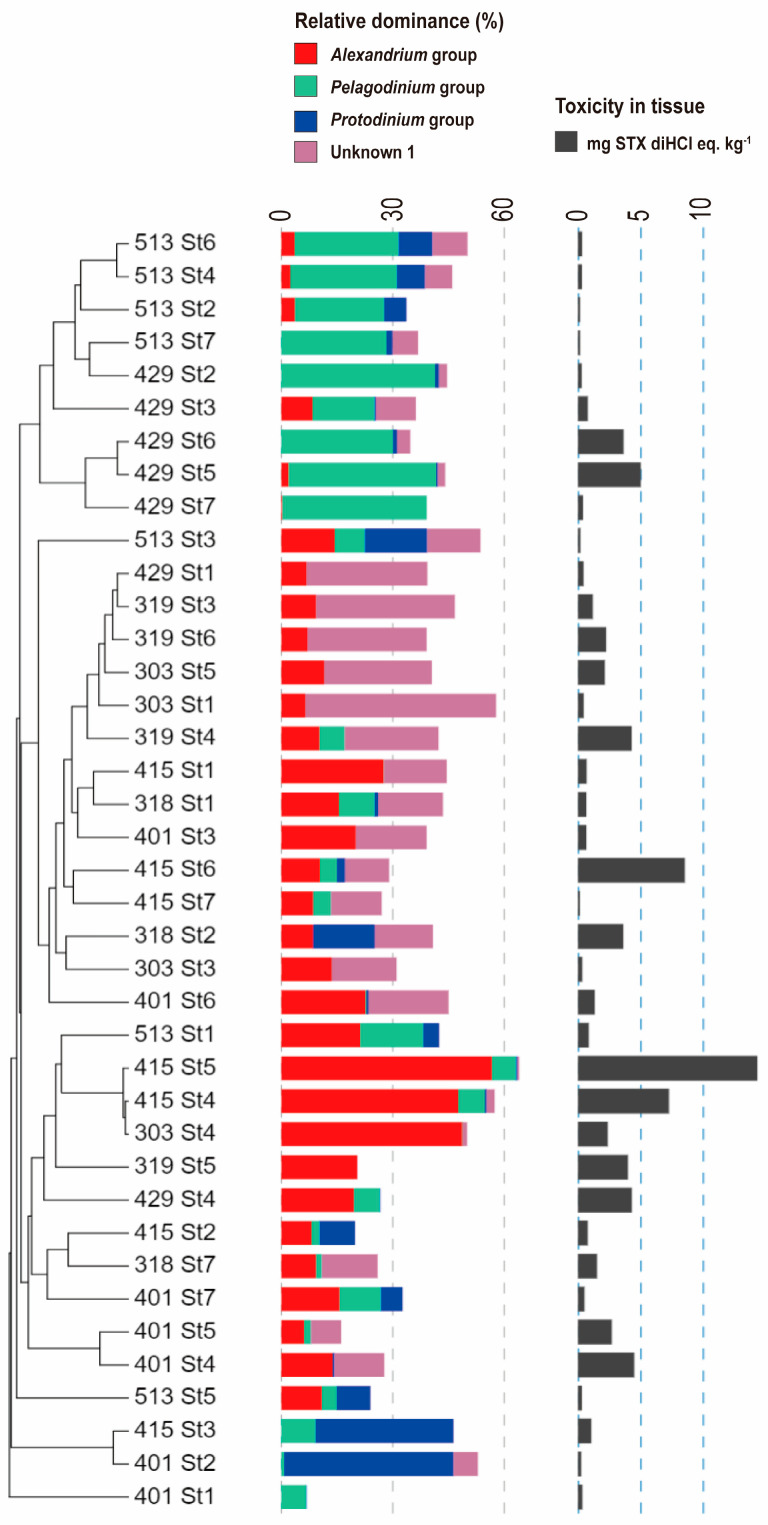
Relative dominance of major dinoflagellate groups (see Figure 5 for grouping) found in guts and PSP toxicity measured in tissues of mussel samples. The clustering of samples y(month−day−station) was conducted based on the composition of dinoflagellate sequences.

The toxicity of PSTs in mussel tissues correlated significantly and positively with the prevalence of the *Alexandrium* group, particularly in samples where *Alexandrium* was more dominant (Pearson correlation test, r = 0.57, *p* < 0.001) (Figure 5).

However, the OTUs labeled as Unknown1 were rarely found in seawater samples but were present in 28 of the 39 mussel gut samples, with a maximum and mean dominance of 51.4% and 11.4% (SD of 12.1%), respectively (Figure 4 and Figure 5).

## 3. Discussion

By concurrently examining the toxicities of PSTs and toxin profiles in microalgae and mussels in coastal environments, this study gained insights into the ecological processes that lead to PSP outbreaks in temperate coastal areas in spring, when mussel harvesting and sales are frequently prohibited owing to elevated PST toxicity. The toxicities of the PSTs fluctuated over an extremely wide range. Despite this high variability, the PSP toxins detected in most seawater and mussel tissue samples were predominantly combinations of C1/2, GTX1, and GTX4. However, the relative proportions of these toxins varied greatly depending on the location and time of the survey, indicating a high degree of spatiotemporal variability in the toxin composition. In addition, the analysis of dinoflagellate diversity in mussel guts clearly showed that *Alexandrium* species play a major role in shellfish toxin enrichment. However, in some samples, smaller microalgae capable of passing through the 20 μm net may have contributed to toxin accumulation.

**Figure 6:**
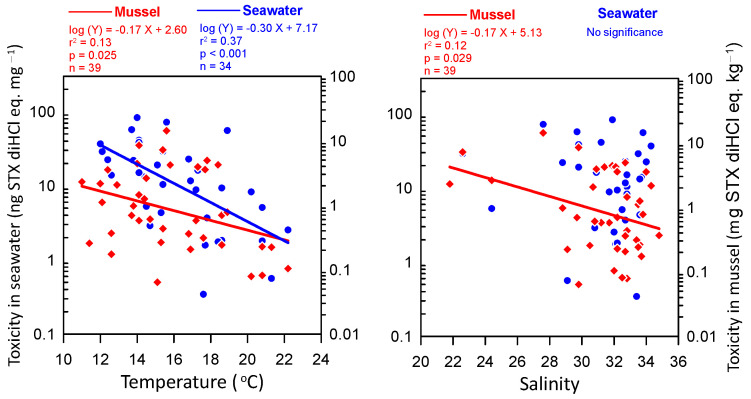
Relationship between seawater and mussel toxicity and temperature and salinity. The blue and red symbols represent seawater and mussel samples, respectively. The solid lines represent regression lines, and their statistical parameters are shown above the graph.

Recent studies have shown notable spatiotemporal variability in PSP toxin concentrations and profiles in marine microalgae and shellfish [20,22,23,27]. The amounts and types of toxins present in toxic microalgae are influenced by several factors. Environmental factors such as water temperature, salinity, nutrient availability, and N/P ratio are known to affect PSP toxin amounts and profiles [9,23,24,26,27,28,29]. A previous study conducted in Jinhae Bay showed that PSP toxin concentrations were affected by water temperature in the range of 12–19 °C [23,30,31]. Consistent with our study, most previous studies identified peak toxicity in mid-April at a seawater temperature of ~15 °C [30,31,32]. However, Mok et al. [23] identified peak toxicity at the beginning of May, but the seawater temperatures in their study were relatively low and reached approximately 15 °C at the beginning of May. Therefore, seawater temperature likely plays a pivotal role in determining the timing of peak PSP toxicity in the study area. Another study at the same site suggested that the influx of river water and the subsequent increase in nitrogen nutrient concentrations may trigger the growth of *A. catenella*, increasing the number of toxin-producing organisms in the seawater [22]. Given the large fluctuations in water temperature and salinity among the stations in this study (Figure 1), variations in water temperature and salinity have an obvious influence on the variability of microalgae and toxin content in mussels, although subsequent changes in nutrient conditions may also affect this variability. In fact, PST toxicity in both seawater and mussel tissue demonstrated a negative correlation with seawater temperature, with maximum toxicities occurring at approximately 15 °C (Figure 6). In addition, a significant negative relationship was observed between salinity and mussel toxicity (Figure 6). However, despite the significant relationships with temperature and salinity, the low coefficient of determination (r^2^) of these regressions suggests that spatiotemporal variations in toxicity may be more strongly influenced by ecological and physiological factors, such as nutrients and toxic algae composition, and biological mechanisms, such as toxin accumulation and depurination (see below). Shellfish toxin profile studies conducted in the spring near Jinhae Bay since 1987 have consistently demonstrated that GTX1 and GTX4 are the most predominant toxins, as observed in this study [22,23,30,32]. This result suggests that a similar mechanism has driven shellfish poisoning in this region for approximately 30 years.

In addition to these environmental factors, changes in the composition of toxic microalgae may be responsible for the spatiotemporal variability of these PSTs [9,24,33]. A high diversity of dinoflagellates (153 OTUs with sequence occurrence greater than 1%) was observed in the samples of this study. The dominance of sequences belonging to the *Alexandrium* clade, a well-known PSP-causing toxic microalga, was evident in most samples (Figure 4). Furthermore, the high proportion of *Alexandrium* in samples with particularly high PST levels (Figure 5) in this study underscores that *Alexandrium* species significantly contribute to the PST toxicity in the mussel samples. Studies conducted in Jinhae Bay revealed a similar trend in *Alexandrium* abundance and PSP toxicity, with a lag of a few days [22,30]. A similar trend was also observed along the west coast of South Africa [27]. Thus, toxicity in mussels seems to correspond to that in seawater, with a lag of several days. Moreover, the temporal changes in toxicity in both the seawater and mussel samples in this study from the inner bay stations, which presented relatively high toxicity, showed some correspondence (Figure 3 and Figure 4). However, in seven mussel samples with toxicities ranging from 0.1 to 3.6 mg STX diHCl eq. kg^−1^, *Alexandrium* group sequences were rare and accounted for a fraction of less than 1% (Figure 5); thus, contributions from other unknown microalgae to PSP toxicity in mussels cannot be ruled out. Notably, in several highly toxic mussel gut samples, “Unknown1” OTUs were the most dominant dinoflagellate; however, in the seawater samples, this dinoflagellate was not found (Figure 4 and Figure 5). This finding suggests the presence of PST-causing dinoflagellates smaller than 20 μm that were not captured by the phytoplankton nets. A study conducted in the St. Lawrence Estuary showed that plankton below 15 μm, which excludes *Alexandrium*, accounted for up to 47% of the PSTs in seawater [21]. Similarly, a study conducted in Tanabe Bay, Japan, also showed higher toxin levels in the 5–20 μm particle fraction than in the >20 μm fraction containing *Alexandrium* [26], suggesting that small plankton other than *Alexandrium* may be responsible for PSTs.

A positive correlation (r = 0.65, *p* = 0.013, n = 14) between the toxicities in seawater and mussels was found in the inner bay station with relatively high peak toxicities. Nonetheless, variations in toxicity in the seawater samples were not consistent with those in the mussel samples. Thus, the transfer and accumulation of toxins from seawater to mussels is unlikely to be synchronized and probably depends on a variety of factors. Since the toxin concentration and toxic algal abundance were not quantified based on seawater volume in this study, direct comparisons between seawater and mussel toxicity may have limited practical value. However, given that toxin profiles also exhibited notable differences between the two sample types (Figure 2 and Figure 3), it seems unlikely that such discrepancies can be attributed solely to methodological limitations. One potential explanation for the lack of correlation between the toxin concentrations in the two samples is the disparate temporal scales of the processes that gave rise to the observed toxin concentrations at the time of sampling. The concentrations of toxins in the mussels reflect their accumulation and/or elimination over a period of days to weeks [1,34,35]. In contrast, the toxin concentrations in the seawater and the composition of toxic microalgae that cause different toxin profiles [36,37] fluctuate over a period of days or even less. In addition to the amount of toxins, differences were observed in the toxin composition between the two types of samples. Although GTX1 and GTX4 were the predominant toxins in both seawater and mussel tissues, certain toxins were detected in only one type of sample. In addition to the above explanation, mussels can transform the ingested toxins into different forms that may alter their toxicity. This biological defense mechanism may serve to protect mussels against the adverse effects of PSP toxins [27,34]. Consequently, the bioconversion of toxins after ingestion may reinforce these differences. Notably, in the present study, only mussel samples with low PSP toxicity exhibited high relative toxicities to relatively minor and diverse toxins, such as dcSTX and dcGTX3 (Figure 3). The bioconversion of C1/2 to dcGTX2/3 has been reported in bivalves [38]. Similarly, in a *Gymnodinium catenatum* feeding study, dcSTX and dcGTX2/3 were present at high levels in clams [39]. It was postulated that this enrichment was due to the fact that dcSTX produced by post-feeding transformation has a slower depuration rate relative to that of other toxins, and therefore remains in shellfish for a relatively long period. In this context, the high proportion of these toxins observed in some mussel samples suggests that these samples may have been in the later stages of the depuration phase when the additional uptake of toxins was reduced. Conversely, the proportion of the sum of highly toxic toxins (GTX4, GTX1, dcGTX2, dcGTX3, GTX2/3, NEO, dcSTX, and STX) with toxicity equivalency factor (TEF) > 0.7 [4] in this study was as high as 80% in both seawater samples and mussel tissues, with no statistical difference between the two samples (paired *t*-test, *p* > 0.05). This suggests that the continuous supply of toxic microalgae from seawater during spring outweighs the transformation of these toxins into less toxic forms in the mussel gut.

## 4. Conclusions

The results of this extensive analysis of PST concentrations in both seawater and mussels and evaluation of dinoflagellate community composition along the southern coast of Korea underscore the significant spatiotemporal variability in PST levels, highlight the predominant role of *Alexandrium* species and indicate the possible contribution of other unknown toxic algae to PST dynamics within the region. Water temperature and salinity were identified as factors that influence PST production and shellfish poisoning in this study. The spatiotemporal patterns observed in the PST concentrations and dinoflagellate community composition suggest complex environmental factors influence PST dynamics. To better understand PSP outbreaks in coastal areas, further quantitative research is needed on how the interactions among the environment, toxic algae, and shellfish impact PST dynamics.

## 5. Materials and Methods

### 5.1. Study Areas and Sampling

**Figure 7:**
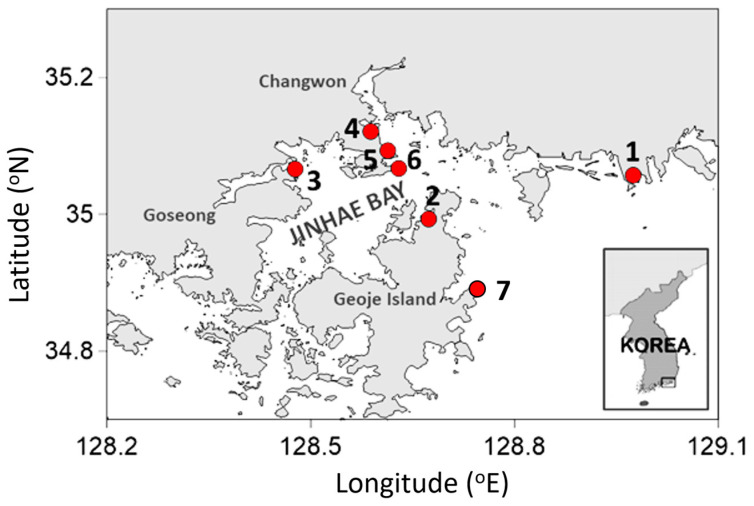
Map showing the study area and stations. The numbers represent the names of the stations.

Each survey was conducted at 4 to 7 stations (Stn) along the southern coast of South Korea (Figure 7). Stns 4, 5, and 6 were located in the inner bay, where mussel farming is active. Six surveys were conducted at approximately 2-week intervals from early March to mid-May when mussel harvesting is often prohibited because of high PST toxicity above the allowance criteria (0.8 mg STX diHCl eq. kg^−1^). Seawater samples were collected using a 20 μm phytoplankton net, which was dragged 5–10 times to collect an approximately 1.5 L net sample. At the same time, approximately 10 mussels were collected from among naturally growing individuals around the seawater collection sites. For the PST concentration analysis in the seawater, approximately 500 mL of the net sample was immediately filtered through a GF/F filter in the field to concentrate the microalgae. For DNA analysis, approximately 200 mL of the net sample was filtered through a 0.2 μm polycarbonate filter and preserved in STE buffer (100 mM NaCl, 10 mM EDTA, 10 mM Tris-HCl, pH 8.0). The samples pretreated in the field were transported on ice to the laboratory and, upon arrival, were stored in a deep freezer at −70 °C. The mussel (*Mytilus galloprovincialis*) samples were also transported to the laboratory on ice and stored in the same manner. The tissue and guts were collected separately and stored in the laboratory for PST and DNA analyses, respectively.

### 5.2. PST Concentration in Microalgae and Mussel Tissues

The net samples collected on the GF/F filter were freeze-dried, and a portion was used to analyze PSP toxins. Mussel samples were prepared according to the Food Public Code provided by the Korean Ministry of Food and Drug Safety, and 5 g of the wet tissue mixture from approximately 10 mussels was used for analysis. PSP toxins in the seawater samples were extracted with 0.03 M acetic acid, centrifuged, and then filtered through a 0.2 μm syringe filter [40]. For the mussel samples, PSP toxins were extracted using 0.1 N HCl, proteins were then precipitated with trichloroacetic acid (TCA), and the solution was then filtered through a 0.2 μm syringe filter, as described in a previous study [41]. The PSP toxin analysis was conducted using an HPLC-FLD (e2695, Waters, Milford, MA, USA) coupled to a post-column reactor module. Certified reference materials (C1/2, GTX1/4, GTX2/3, dcGTX2/3, NEO, dcNEO, STX, and dcSTX) were purchased from the National Research Council (NRC) of Canada (Halifax, NS, Canada) and Cifga (Lugo, Spain). Matrix-matched calibration solutions were prepared for PSP toxin analysis of mussel samples [42]. TEFs recommended by the European Food Safety Authority [4] were used to convert the concentrations of individual toxins into total toxin levels in saxitoxin equivalents.

### 5.3. DNA Extraction and Amplicon Library Construction and Sequencing

Microalgal DNA was collected from the filters using a combination of phenol–chloroform extraction [43] and spin column purification, which was performed following the protocol of the Qiagen Blood & Tissue Kit (Qiagen, Redwood City, CA, USA). DNA from the mussel guts was purified according to the protocol described in the Qiagen Blood & Tissue Kit (Qiagen, Redwood City, CA, USA). The species composition of the dinoflagellates was analyzed from the sequence of the mitochondrial cytochrome b oxidase gene (*cob*). The Dinocob4F-Dinocob3R primer set was used for PCR amplification of the *cob* gene, which is known to be effective in amplifying most dinoflagellate species [44]. For the MiSeq platform, amplicon PCR, PCR clean-up, and index PCR were performed as described in the MiSeq manual [45]. The first PCR was performed as described by Lin et al. [44]. After clean-up of the second PCR using AMPure XP beads (Beckman Coulter Inc., Brea, CA, USA), the final PCR products were quantified using a Nanodrop 1000 spectrophotometer (Thermo Scientific, Waltham, MA, USA). Identical amounts of each product were pooled and sequenced on an Illumina MiSeq 2 × 300 PE platform at CJ BioScience (Seoul, Republic of Korea).

### 5.4. Sequence Data Analyses

Sequence reads were processed using Mothur software (version 1.48), as suggested by the MiSeq standard operating procedures, with some modifications [46]. Any contigs with ambiguous bases (N) or those longer or shorter than 385 bp were removed. A reference database for alignment and classification was created using 258 *cob* genes of dinoflagellates retrieved from GenBank (https://www.ncbi.nlm.nih.gov/genbank/; accessed on 5 July 2022; [47]). A more detailed method for processing the sequences was described by Choi et al. [48]. During the analysis, reads with a sequence identity of >99.5% were clustered into OTUs. After classification, the OTUs belonging to Dinophyceae were selected for further analysis. In total, 839,185 dinoflagellate sequence reads were obtained from 25 seawater and 39 mussel gut samples. The number of reads per sample varied significantly, ranging from 1795 to 61,377. Therefore, the reads were normalized to the lowest number of reads—1795. A reference phylogenetic tree of the translated amino acid sequences of the *cob* gene sequences was constructed using the maximum-likelihood method in the *raxml* program [49] and the PROTGAMMAAUTO model with 100 bootstrap replicates. The translated representative sequences obtained in this study were added to the reference tree using the maximum parsimony option in the ARB program [50].

### 5.5. Other Analyses

The seawater temperature and salinity were measured using a handheld meter (Pro 2030, YSI Incorporated, Yellow Springs, OH, USA). A heat map was drawn using the iTOL program [51]. A cluster analysis of the samples according to dinoflagellate composition was conducted using Primer software (version 7) [52]. In the analysis, the relative percentages of major OTUs (those with >5% dominance) were log(x + 1) transformed, and the samples were clustered based on the Bray–Curtis resemblance matrix. Pearson correlation analyses and *t*-tests were conducted using SPSS Statistics 26.0 (IBM, Armonk, NY, USA).

## Data Availability

The data of cob gene sequences presented in this study are available on NCBI (https://www.ncbi.nlm.nih.gov/bioproject/PRJNA1112587; accessed on 26 June 2024).
